# Multi-vendor evaluation of artificial intelligence as an independent reader for double reading in breast cancer screening on 275,900 mammograms

**DOI:** 10.1186/s12885-023-10890-7

**Published:** 2023-05-19

**Authors:** Nisha Sharma, Annie Y. Ng, Jonathan J. James, Galvin Khara, Éva Ambrózay, Christopher C. Austin, Gábor Forrai, Georgia Fox, Ben Glocker, Andreas Heindl, Edit Karpati, Tobias M. Rijken, Vignesh Venkataraman, Joseph E. Yearsley, Peter D. Kecskemethy

**Affiliations:** 1 The Leeds Teaching Hospital NHS Trust, Leeds, UK; 2 Kheiron Medical Technologies, London, UK; 3 Nottingham Breast Institute, City Hospital, Nottingham University Hospitals NHS Trust, Nottingham, UK; 4 MaMMa Egészségügyi Zrt, Budapest, Hungary; 5 Duna Medical Center, Budapest, Hungary; 6 GÉ-RAD Kft, Budapest, Hungary; 7 Department of Computing, Imperial College London, London, UK; 8 Medicover, Budapest, Hungary

**Keywords:** Breast cancer screening, digital mammography, artificial intelligence, generalisability

## Abstract

**Background:**

Double reading (DR) in screening mammography increases cancer detection and lowers recall rates, but has sustainability challenges due to workforce shortages. Artificial intelligence (AI) as an independent reader (IR) in DR may provide a cost-effective solution with the potential to improve screening performance. Evidence for AI to generalise across different patient populations, screening programmes and equipment vendors, however, is still lacking.

**Methods:**

This retrospective study simulated DR with AI as an IR, using data representative of real-world deployments (275,900 cases, 177,882 participants) from four mammography equipment vendors, seven screening sites, and two countries. Non-inferiority and superiority were assessed for relevant screening metrics.

**Results:**

DR with AI, compared with human DR, showed at least non-inferior recall rate, cancer detection rate, sensitivity, specificity and positive predictive value (PPV) for each mammography vendor and site, and superior recall rate, specificity, and PPV for some. The simulation indicates that using AI would have increased arbitration rate (3.3% to 12.3%), but could have reduced human workload by 30.0% to 44.8%.

**Conclusions:**

AI has potential as an IR in the DR workflow across different screening programmes, mammography equipment and geographies, substantially reducing human reader workload while maintaining or improving standard of care.

**Trial registration:**

ISRCTN18056078 (20/03/2019; retrospectively registered).

**Supplementary Information:**

The online version contains supplementary material available at 10.1186/s12885-023-10890-7.

## Introduction

Despite continuous improvements in therapy, breast cancer remains the leading cause of cancer-related mortality among women worldwide, accounting for approximately 600,000 deaths annually [1]. Randomised trials and incidence-based mortality studies have demonstrated that population-based screening programs substantially reduce breast cancer mortality [2–6].

Full-field digital mammography (FFDM) is the most widely used imaging modality for breast cancer screening globally [7–11]. Using two readers (double reading), with arbitration, is shown to increase cancer detection rates by 6–15%, while keeping recall rates low [12–14]. The model is standard practice in over 27 countries in Europe, and in Japan, Australia, the Middle East and the UK [8–11]. However, the high cost of two expert readers to interpret every mammogram, alongside growing shortages of qualified readers, means double reading is difficult to sustain [15–17].

In the past, computer-aided detection (CAD) software have been used to automate some screening mammogram analysis, which has been adopted by over 83% of US facilities [18], but recent studies questioned CAD’s benefit to screening outcomes [19, 20]. When tested in the United Kingdom National Health Service Breast Screening Programme (UK NHSBSP) as an alternative to double reading, a traditional CAD system reduced specificity with a significant increase in recall rates [21].

Modern artificial intelligence (AI) technologies have emerged as a promising alternative. Recent studies suggest the current generation of AI-based algorithms using convolutional neural networks may interpret mammograms at least to the level of human readers [22–26]. These included small-scale reader studies [22–25, 27] and larger-scale retrospective studies [25, 26, 28, 29] often performed on artificially enriched datasets, involving resampling [22–27, 29, 30], to approximate screening prevalence but without covering the full distribution of cases expected in screening populations. Many of these studies were skewed to one mammography equipment vendor, raising questions about the true generalisability of the AI systems tested. The potential of AI to positively transform clinical practice in real-world screening remains to be confirmed, as also highlighted in a recent systematic review [31]. Concerns remain about AI’s generalisation across heterogeneous deployment environments with different population groups and mammography equipment vendors. Previous studies lack sufficient evidence of multi-vendor generalisation of AI which is a critical requirement for safe and effective deployment.

Rigorous large-scale, multi-vendor studies are needed to assess performance of AI in double reading on diverse cohorts of women across multiple screening sites and programmes, and on unenriched screening data representative of populations the AI will process in real-world deployments. Such studies should evaluate model performance on images from various vendors of mammography equipment, using clinically relevant screening metrics. This study aimed to evaluate whether an AI system could act as a reliable independent reader, generalising across a range of mammography equipment while automating a substantial part of the double reading workflow.

## Methods

### Study design

We first evaluated simulated double reading performance using AI compared to historical human double reading to assess the generalisability of using the AI as an independent second reader across different mammography equipment. In addition, the AI system’s standalone performance is reported to provide a more comprehensive assessment of the AI’s consistency across different mammography equipment. The historical first human reader’s performance is also reported to provide context as the only guaranteed independent read at all participating sites.

All comparisons were determined on the same unenriched cohorts. Performance was measured in terms of sensitivity, specificity, recall rate, cancer detection rate (CDR), positive predictive value (PPV), and arbitration rate (rate of disagreement between the first and second readers) (see Supplement, Sect. 1).

The statistical analysis plan was developed and executed by an external Clinical Research Organisation (CRO) (Veristat LLC, supported by Quantics Consulting Ltd). All results presented for the listed metrics are CRO-verified. Other results presented are results of post hoc analyses.

The study had UK National Health Service (NHS) Health Research Authority (HRA) (reference: 19/HRA/0376) and ETT-TUKEB (Medical Research Council, Scientific and Research Ethics Committee, Hungary) approval (reference: OGYÉI/46651–4/2020).

### Study population and cohorts

**Fig. 1:**
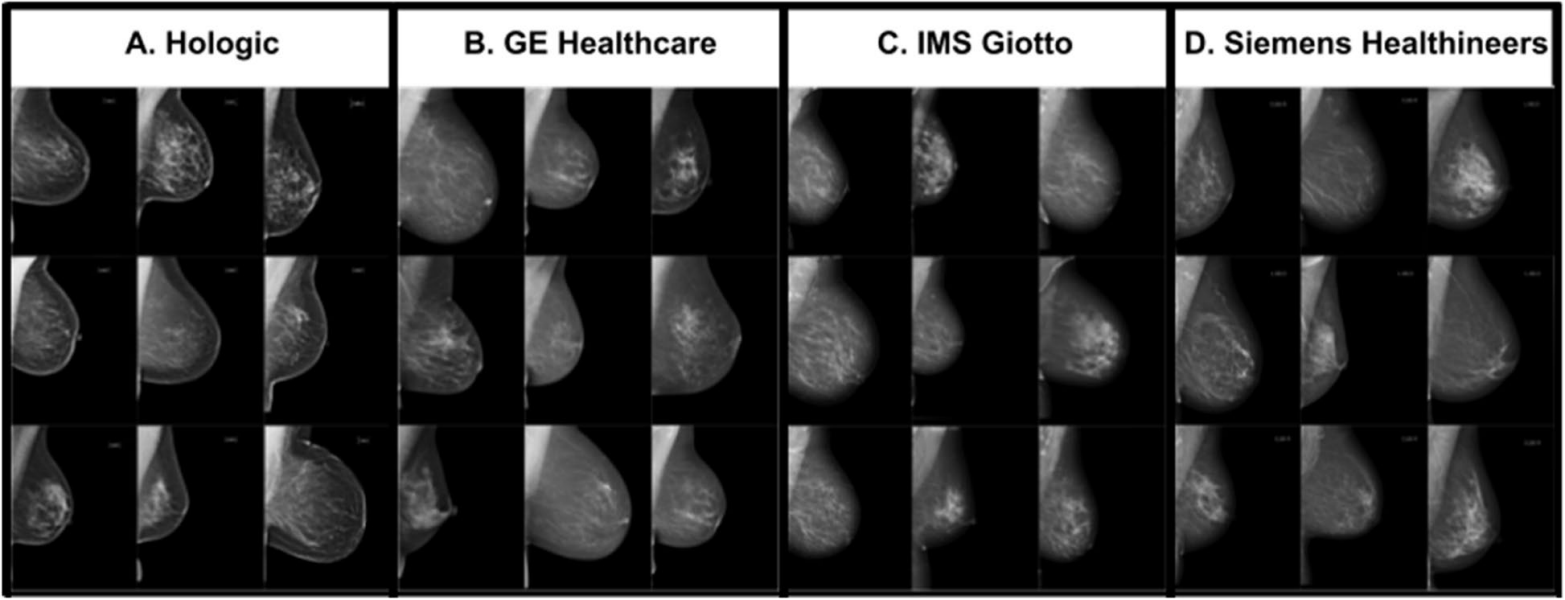
Four mammography vendors of image acquisition equipment are included in this evaluation: **A** Hologic, **B** GE Healthcare, **C** IMS Giotto, and **D** Siemens Healthineers. A few example images taken by each vendor’s mammography equipment are presented. Images produced by mammography equipment vendors A through D range in how much sharp versus soft features are emphasized with **A **emphasizing sharp features the most and **D** emphasizing softer features the most

All analyses were conducted on a historical cohort of de-identified cases from seven European sites representing four centers: three from the UK and one in Hungary (HU), between 2009 and 2019. The three UK centers included Leeds Teaching Hospital NHS Trust (LTHT), Nottingham University Hospitals NHS Trust (NUH), and United Lincolnshire Hospitals NHS Trust (ULH). All sites participate in the UK NHSBSP overseen by Public Health England (PHE) and adhere to a three-year screening interval, with women between 50 and 70 years old invited to participate. A small cohort of women between 47 and 49 years, and 71 and 73 years old who were eligible for the UK age extension trial (Age X) were also included [32]. The Hungarian center, MaMMa Klinika (MK), involved four sites and their mobile screening units, which follow a two-year screening interval and invite women aged 45 to 65. Across all sites, women outside the regional screening programme age range, who chose to participate as per standard of care (opportunistic screening) were also included. Screening cases were acquired from the dominant mammography equipment vendor at each site: Hologic (at LTHT), GE Healthcare (NUH), Siemens Healthineers (ULH), and IMS Giotto (MK) (Fig. 1).

**Fig. 2:**
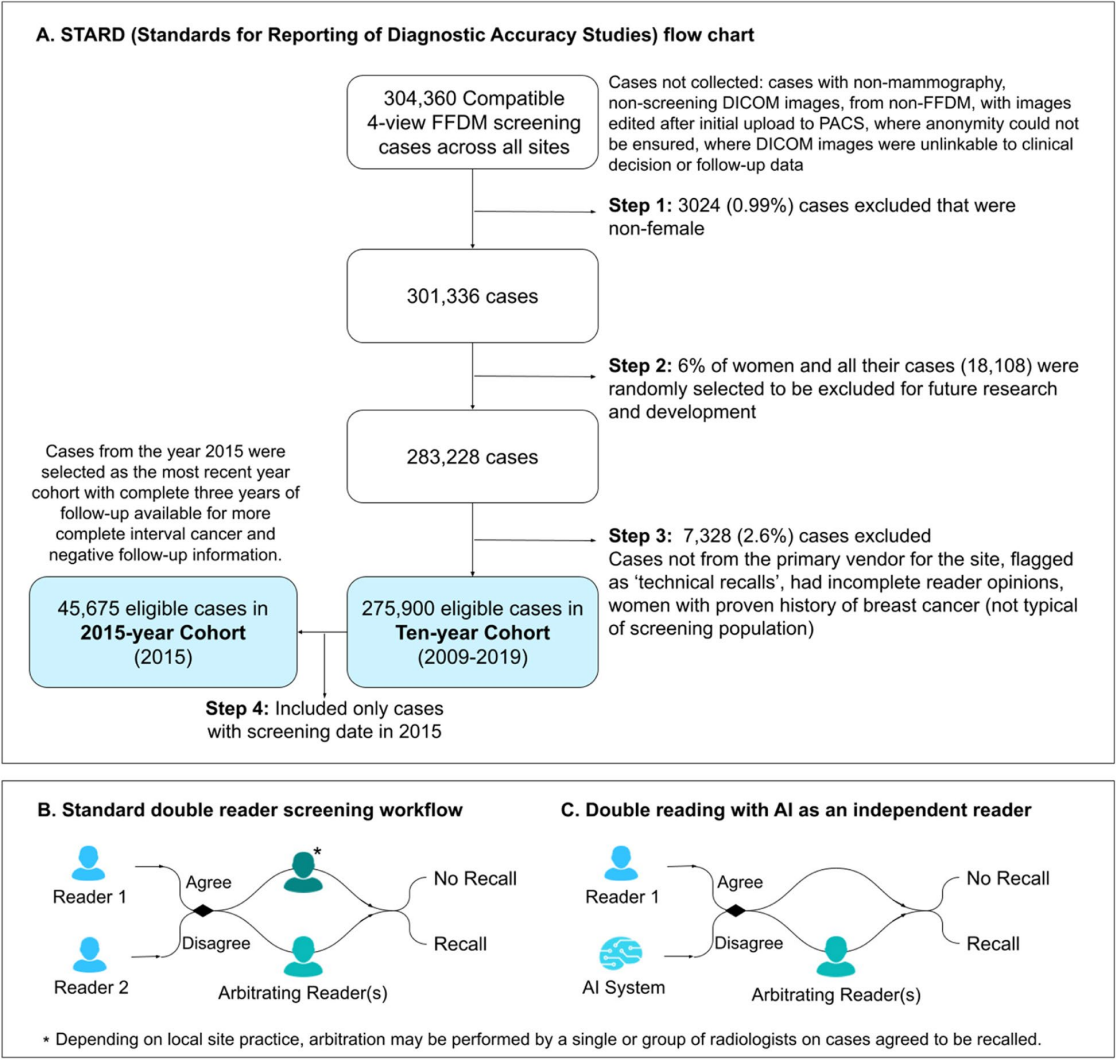
**A** STARD (Standards for Reporting of Diagnostic Accuracy Studies) flow chart describing case eligibility and the final two study cohorts, ‘ten-year’ and ‘2015-year’. **B **Standard double reader screening workflow. **C** Double reading with AI as an independent reader

In total, 304,360 cases were extracted which were compatible 4-view FFDM screening cases. Cases were excluded in three steps, including the exclusion of all cases from a random 6% of women and the exclusion of cases that the AI would not process in real-world deployments (Fig. 2A). A set of 275,900 eligible cases resulted, from which two unenriched, representative cohorts were created: one from the whole ten-year period (2009–2019) including 275,900 eligible cases from 177,882 participants, and one for a single year (2015) including 45,675 eligible cases (Fig. 2A). The ‘ten-year’ cohort included screening activity over the whole ten years and any follow-up information available. The ‘2015-year’ cohort included a complete three years of follow-up and thus was used for further analysis as a cohort with more complete three-year IC information and three-year negative follow-up information. Multiple cases were allowed per participant in both cohorts, but 99.98% of participants had only one case in the 2015-year cohort.

### Standard of care double reading and double reading with an AI system

At all sites, the historical first reader's opinion was made in isolation, and the second reader had access, at their discretion, to the opinion of the first. In cases of disagreement, an arbitration, performed by a single or group of radiologists, determined the final “recall” or “no recall” decision. When the first and second reader opinions agreed “no recall”, a “no recall” decision was reached. When the opinions agreed “recall”, then either a “recall” decision was reached, or an arbitration performed by a single or group of radiologists made the definitive “recall” or “no recall” decision, depending on the site’s local practice (Fig. 2B).

Double reading with the AI system was simulated by combining the opinion of the historical first reader with the AI system (Fig. 2C). When both agreed, a definitive “recall” or “no recall” decision was made. Upon disagreement, if available, the historical arbitration opinion was used, otherwise the historical second reader opinion, which happens to agree with the historical first reader opinion in such circumstances, was applied to simulate the arbitration process.

### AI System

All study cases were analysed by the Mia™ version 2.0 'AI system', developed by Kheiron Medical Technologies (London, UK). The underlying technology of the AI system is based on deep convolutional neural networks (CNNs), the state-of-the-art machine learning methodology for image classification. The AI system works with standard DICOM (Digital Imaging and Communications in Medicine) cases as inputs, analyses four images with two standard FFDM views per breast, and generates a binary suggestion of "recall" (for further assessment due to suspected malignancy) or “no recall” (until the next screening interval). The AI system’s output is deterministic, and is based on a single prediction per case. The system used pre-defined decision thresholds for “recall” or “no recall”, which were set using data from patients and cases not included in the study. They were set to be at a balanced sensitivity and specificity when screen-detected cancers only are included in calculating sensitivity.

The AI software version was fixed prior to the study. All study data came from participants whose data was never used in any aspect of algorithm development and was separated from and inaccessible for research and development.

### Determining ground truth, subsample definitions and metrics

Sensitivity, cancer detection rate (CDR), and positive predictive value (PPV) were calculated with positives defined as ‘screen-detected positives’ and ‘interval cancers (ICs)’, collectively. Screen-detected positives were screening cases correctly identified by the historical double reader workflow, with a pathology-proven malignancy confirmed by fine needle aspiration cytology (FNAC), core needle biopsy (CNB), vacuum-assisted core biopsy (VACB), and/or histology of the surgical specimen within 180 days of the screening exam. Three-year and two-year ICs were considered for the UK and HU, respectively, corresponding with their screening interval periods. Three-year and two-year ICs were defined as a screening case with a pathology-proven cancer arising within 1,095 days or 730 days, respectively, following the original screening date. For the UK sites, ground truth for malignancy was obtained via the NHS National Breast Screening Service (NBSS) database including cancer registry information. In Hungary, confirmation of malignancy was obtained from digital pathology reports in patient health records.

Specificity was calculated on negatives defined as any screening case with evidence of a ‘three-year’ negative follow-up result that includes a mammography reading at least 1,035 days (i.e. two months less than a three-year screening interval) after the original screening date, with no proof of malignancy in between.

Recall rate, CDR, and arbitration rate were calculated on the whole population, which included confirmed positives, confirmed negatives, as well as unconfirmed cases (neither confirmed positive nor negative) to assess performance on a real-world screening case distribution.

### Statistical methods

Two-sided 95% confidence intervals (CIs) were reported for all metrics. For non-inferiority and superiority testing, ratios of proportions were used to calculate relative differences. The non-inferiority margin was set at 10%. For metrics where a higher result indicates better performance, this meant that non-inferiority was concluded if the ratio was > 0.9. As the non-inferiority tests used only one side of the 95% CIs, the one-sided alpha was 2.5%. The 10% margin has been previously used for the assessment of mammography screening with CAD systems, but the 97.5% non-inferiority confidence is stricter than the 90 to 95% commonly used [18, 21].

Superiority was tested when non-inferiority was passed, with the same confidence intervals and alpha. For superiority testing to pass, the lower bound of the confidence interval of the ratio had to be above 1.

## Results

### Study population and reading workflow

Table 1 presents characteristics of the study population. Of the 275,900 total cases, there were 2792 (1.0%) positives overall, of which, 2310 (0.84%) were screen-detected positives. The observed proportion of ICs was 0.14% in the ten-year cohort and 0.20% in the 2015-year cohort, where IC data was more complete.

**Table 1:** Characteristics of ten-year and 2015-year cohorts

Characteristics		Ten-year cohort (2009–2019)		2015-year cohort (2015)	
		**Number of cases**	**Proportion of study population**	**Number of cases**	**Proportion of study population**
**Total**^**a**^		275,900	100.0%	45,675	100.0%
Center / Vendor	MK / IMS Giotto	83,410	30.2%	10,462	22.9%
	NUH / GE	69,045	25.0%	10,983	24.0%
	LTHT / Hologic	64,645	23.4%	10,717	23.5%
	ULH / Siemens	58,800	21.3%	13,513	29.6%
Age	< 40	483	0.2%	5	< 0.1%
	40—49	37,696	13.7%	5,575	12.2%
	50—59	114,524	41.5%	19,399	42.5%
	60—69	98,289	35.6%	16,772	36.7%
	70—79	23,359	8.5%	3,702	8.1%
	80—89	1,534	0.6%	221	0.5%
	> 90	15	< 0.1%	1	< 0.1%
Positives	Total positives^b^	2,683	0.97%	457	1.00%
	Screen-detected positives	2,310	0.84%	365	0.80%
	Interval cancers (ICs)	373	0.14%	92	0.20%
	Three-year ICs from UK	289	0.10%	80	0.18%
	Two-year ICs from HU	84	0.03%	12	0.03%

^a ^Total number of cases for which CDR, recall rate, and arbitration rate were calculated on

^b^ Used for sensitivity, CDR, and PPV calculations

### Multi-vendor performance in the double reading workflow

The performance of double reading with AI was estimated using a simulation with historical reader opinions. The statistical tests showed that double reading with the AI system compared to historical double reading for each mammography equipment vendor and site was at least non-inferior at every metric, with superiority tested and passed for recall rate, specificity and PPV for some vendors and sites (Table 2). Table S2 in the supplement presents the number of confirmed negative, confirmed positive, and unconfirmed cases recalled and not recalled by double reading with and without AI by site and mammography equipment vendor. Regional results are presented in Table 3, assessing generalisation across geographies.

**Table 2:** Performance of double reading with and without AI – by site and mammography equipment vendor

**A) MK / IMS Giotto**^b^			
**Performance Metric**	**Historical double reading**	**Double reading (DR) with AI**	**Test outcome for DR with AI**^a^
On ten-year cohort			
Recall rate	9.2% (9.0, 9.4)	7.8% (7.7, 8.0)	**Superior** 0.85 (0.85, 0.86)
CDR	7.7 per 1000 (7.1, 8.3)	7.6 per 1000 (7.0, 8.2)	**Non-inferior** 0.99 (0.98, 0.99)
Sensitivity	88.8% (86.2, 90.9)	87.5% (84.9, 89.7)	**Non-inferior** 0.99 (0.98, 0.99)
Specificity	94.7% (94.3, 95.0)	95.8% (95.4, 96.1)	**Superior** 1.01 (1.01, 1.01)
PPV	8.3% (8.1, 8.6)	9.6% (9.4, 9.9)	**Superior** 1.16 (1.14, 1.16)
On 2015-year cohort: with more complete IC data available			
Recall rate	8.5% (8.0, 9.1)	7.5% (7.0, 8.0)	**Superior** 0.88 (0.85, 0.90)
CDR	7.5 per 1000 (6.0, 9.3)	7.4 per 1000 (5.9, 9.2)	**Non-inferior** 0.99 (0.96, 1.00)
Sensitivity	87.6% (79.2, 93.9)	86.5% (77.9., 92.1)	**Non-inferior** 0.99 (0.96, 1.00)
Specificity	95.8% (94.6, 96.7)	96.9% (95.8, 97.6)	**Superior** 1.01 (1.01, 1.02)
PPV	8.7% (7.0, 10.8)	9.8% (7.9, 12.1)	**Superior** 1.12 (1.09, 1.14)
**B) NUH / GE**^c^			
**Performance Metric**	**Historical double reading**	**Double reading (DR) with AI**	**Test outcome for DR** **with AI**^a^
On ten-year cohort			
Recall rate	2.8% (2.7, 2.9)	2.8% (2.7, 3.0)	**Non-inferior** 1.01 (0.99, 1.03)
CDR	8.8 per 1000 (8.1, 9.5)	8.6 per 1000 (7.9, 9.3)	**Non-inferior** 0.98 (0.96, 0.99)
Sensitivity	85.5% (82.7, 87.9)	83.5% (80.6, 86.1)	**Non-inferior** 0.98 (0.96, 0.99)
Specificity	97.9% (97.7, 98.1)	97.9% (97.7, 98.1)	**Non-inferior** 1.00 (0.9995, 1.00)
PPV	31.6% (29.5, 33.7)	30.4% (28.4, 32.5)	**Non-inferior** 0.96 (0.95, 0.98)
On 2015-year cohort: with more complete IC data available			
Recall rate	2.8% (2.5, 3.2)	2.8% (2.5, 3.1)	**Non-inferior** 0.99 (0.95, 1.04)
CDR	8.0 per 1000 (6.5, 9.9)	7.9 per 1000 (6.4, 9.8)	**Non-inferior** 0.99 (0.96, 1.00)
Sensitivity	73.9% (65.4, 81.0)	73.1% (64.5, 80.3)	**Non-inferior** 0.99 (0.96, 1.00)
Specificity	98.0% (97.7, 98.3)	98.1% (97.8, 98.4)	**Non-inferior** 1.00 (0.9996, 1.0)
PPV	28.3% (23.6, 33.5)	28.2% (23.5, 33.5)	**Non-inferior** 1.00 (0.97, 1.01)
**C) LTHT / Hologic**^c^			
**Performance Metric**	**Historical double reading**	**Double reading (DR) with AI**	**Test outcome for DR** **with AI**^a^
On ten-year cohort			
Recall rate	5.1% (4.9, 5.3)	5.1% (4.9, 5.2)	**Non-inferior** 0.99 (0.98, 1.01)
CDR	8.2 per 1000 (7.6, 9.0)	8.0 per 1000 (7.4, 8.8)	**Non-inferior** 0.97 (0.96, 0.99)
Sensitivity	87.1% (84.2, 89.5)	84.8% (81.7, 87.4)	**Non-inferior** 0.97 (0.96, 0.99)
Specificity	95.9% (95.7, 96.2)	96.0% (95.7, 96.3)	**Non-inferior** 1.00 (0.9999, 1.00)
PPV	16.2% (15.0, 17.5)	15.9% (14.7, 17.2)	**Non-inferior** 0.98 (0.97, 1.00)
On 2015-year cohort: with more complete IC data available			
Recall rate	4.3% (4.0, 4.7)	4.1% (3.8, 4.5)	**Superior** 0.95 (0.92, 0.99)
CDR	7.7 per 1000 (6.2, 9.5)	7.6 per 1000 (6.1, 9.4)	**Non-inferior** 0.99 (0.96, 1.00)
Sensitivity	88.2% (80.1, 93.3)	87.1% (78.8, 92.5)	**Non-inferior** 0.99 (0.96, 1.00)
Specificity	96.5% (96.0, 96.9)	96.6% (96.1, 97.1)	**Non-inferior** 1.00 (0.9998, 1.00)
PPV	17.7% (14.5, 21.5)	18.3% (15.0, 22.2)	**Superior** 1.04 (1.01, 1.05)
**D) ULH / Siemens**^c^			
**Performance Metric**	**Historical double reading**	**Double reading (DR) with AI**	**Test outcome for DR** **with AI**^a^
On ten-year cohort			
Recall rate	3.6% (3.5, 3.8)	3.6% (3.4, 3.7)	**Superior** 0.98 (0.96, 0.9981)
CDR	9.3 per 1000 (8.6, 10.1)	9.1 per 1000 (8.4, 9.9)	**Non-inferior** 0.97 (0.96, 0.99)
Sensitivity	85.6% (82.7, 88.1)	83.4% (80.4, 86.1)	**Non-inferior** 0.97 (0.96, 0.99)
Specificity	97.4% (97.0, 97.7)	97.5% (97.1, 97.8)	**Non-inferior** 1.00 (0.9994, 1.00)
PPV	25.7% (23.9, 27.6)	25.6% (23.7, 27.5)	**Non-inferior** 1.00 (0.98, 1.01)
On 2015-year cohort: with more complete IC data available			
Recall rate	3.4% (3.1, 3.7)	3.4% (3.1, 3.7)	**Non-inferior** 0.98 (0.95, 1.02)
CDR	9.0 per 1000 (7.5, 10.7)	8.8 per 1000 (7.4, 10.5)	**Non-inferior** 0.98 (0.96, 1.00)
Sensitivity	77.6% (70.4, 83.4)	76.3% (69.0, 82.3)	**Non-inferior** 0.98 (0.96, 1.00)
Specificity	97.6% (97.1, 98.0)	97.7% (97.2, 98.1)	**Non-inferior** 1.00 (0.9988, 1.00)
PPV	26.2% (22.4, 30.4)	26.2% (22.3, 30.4)	**Non-inferior** 1.00 (0.97, 1.02)

95% confidence intervals are presented in parentheses

^a^The ratio of proportions and 95% confidence intervals for assessing non-inferiority and superior are presented

^b^The positive pool for CDR, sensitivity, and PPV include screen-detected positives and two-year ICs, which are relevant for HU

^c^The positive pool for CDR, sensitivity, and PPV include screen-detected positives and three-year ICs, which are relevant for the UK

**Table 3:** Performance of double reading with and without AI by region for the ten-year cohort

**Performance metric**	**Historical double reading**	**Double reading (DR)** **with AI**	**Test outcome for DR with AI**^a^
Regional breakdown for UK^b^			
Recall rate	3.8% (3.8, 3.9)	3.8% (3.7, 3.9)	**Non-inferior** 0.99 (0.98, 1.01)
CDR (3Y)	8.8 per 1000 (8.6, 9.0)	8.6 per 1000 (8.4, 8.7)	**Non-inferior** 0.98 (0.97, 0.98)
Sensitivity (3Y)	86.1% (84.5, 87.6)	83.9% (82.3, 85.6)	**Non-inferior** 0.98 (0.97, 0.98)
Specificity	97.1% (96.9, 97.2)	97.1% (97.0, 97.3)	**Superior** 1.00 (1.00, 1.00)
PPV (3Y)	24.5% (24.0, 25.0)	24.0% (23.5, 24.4)	**Non-inferior** 0.98 (0.97, 0.99)
Regional breakdown for HU^c^			
Recall rate	9.2% (9.0, 9.4)	7.8% (7.7, 8.0)	**Superior** 0.85 (0.85, 0.86)
CDR (2Y)	7.7 per 1000 (7.1, 8.3)	7.6 per 1000 (7.0, 8.2)	**Non-inferior** 0.99 (0.98, 0.99)
Sensitivity (2Y)	88.8% (86.2, 90.9)	87.5% (84.9, 89.7)	**Non-inferior** 0.99 (0.98, 0.99)
Specificity	94.7% (94.3, 95.0)	95.8% (95.4, 96.1)	**Superior** 1.01 (1.01, 1.01)
PPV (2Y)	8.3% (8.1, 8.6)	9.6% (9.4, 9.9)	**Superior** 1.16 (1.14, 1.16)

95% confidence intervals are presented in parentheses

^a^The ratio of proportions and 95% confidence intervals for assessing non-inferiority and superior are presented

^b^The positive pool for CDR, sensitivity, and PPV include screen-detected positives and three-year ICs, which are relevant for the UK

^c^The positive pool for CDR, sensitivity, and PPV include screen-detected positives and two-year ICs only, which are relevant for HU

The comparative performance of double reading with AI compared to historical double reading for cancer detection metrics such as sensitivity, CDR, and PPV are generally improved in the 2015-year cohort where IC data is more complete compared to the ten-year cohort.

Only 24.3% (27/111) of the ICs detected by the AI system were also detected by the double reading simulation with the AI system as the majority of historical readers did not recall the cases. Only 20.9% (37/177) of the cancers historically detected in the next screening round and by the AI system were detected by double reading with the AI system. These portions were lower in the 2015-year cohort, 11.8% (4/34) and 15.1% (8/53), respectively.

Table S4 in the supplement presents the occurrence of cases historically arbitrated or historically not arbitrated when the AI and the historical first reader agree or disagree. Additionally, the performance of the historical arbitrator and historical second reader are presented for the set of cases when the AI and historical first reader disagree.

### Multi-vendor standalone AI performance

While the AI system is not aimed to operate as a standalone reader in clinical practice, assessing the standalone performance characterises the reliability of contribution the AI system could have as an independent reader in the overall double reading workflow across different environments. Results for the standalone AI system and the historical first reader across different mammography equipment and sites are presented in Table 4. Table S3 in the supplement presents the number of confirmed negative, confirmed positive, and unconfirmed cases recalled and not recalled by the AI system and the historical first reader by site and mammography equipment vendor. The performance of the AI system was found to be consistent across different mammography equipment despite their varying image characteristics (see Fig. 1). The AI system’s standalone sensitivity ranged from 76.9% to 85.7% and specificity ranged from 89.2% to 96.1% in the ten-year cohort. Sensitivity ranged from 72.3% to 84.9% and specificity ranged from 89.3% to 96.2% in the 2015-year cohort at the specific operating point assessed. The observed variability in both sensitivity and specificity can be attributed to regional differences. For the UK region with three different types of scanners (Hologic, GE, Siemens), the AI system’s standalone sensitivity ranged from 76.9% to 79.9% and specificity ranged from 89.2% to 89.9% in the ten-year cohort.

**Table 4:** Performance of standalone AI the historical first reader – by site and mammography equipment vendor

**A) MK / IMS Giotto**^a^		
**Performance Metric**	**Historical first reader (%)**	**Standalone AI (%)**
On ten-year cohort		
Sensitivity	79.4 (76.3, 82.2)	85.7 (83.0, 88.1)
Specificity	95.4 (95.0, 95.7)	96.1 (95.7, 96.4)
On 2015-year cohort: with more complete IC data available		
Sensitivity	82.0 (72.8, 88.6)	85.4 (76.6, 91.3)
Specificity	96.5 (95.4, 97.3)	96.2 (95.0, 97.0)
**B) NUH / GE**^**b**^		
**Performance Metric**	**Historical first reader (%)**	**Standalone AI (%)**
On ten-year cohort		
Sensitivity	77.8 (74.6, 80.7)	76.9 (73.7, 79.9)
Specificity	97.3 (97.0, 97.5)	89.6 (89.2, 90.0)
On 2015-year cohort: with more complete IC data available		
Sensitivity	67.2 (58.4, 75.0)	72.3 (63.6, 79.5)
Specificity	97.2 (96.8, 97.5)	90.6 (89.9, 91.3)
**C) LTHT / Hologic**^**b**^		
**Performance Metric**	**Historical first reader (%)**	**Standalone AI (%)**
On ten-year cohort		
Sensitivity	81.0 (77.8, 84.0)	79.9 (76.5, 82.9)
Specificity	95.0 (94.7, 95.3)	89.2 (88.8, 89.6)
On 2015-year cohort: with more complete IC data available		
Sensitivity	82.8 (73.9, 89.1)	84.9 (76.3, 90.8)
Specificity	95.7 (95.1, 96.2)	89.3 (88.5, 90.1)
**D) ULH / Siemens**^**b**^		
**Performance Metric**	**Historical first reader (%)**	**Standalone AI (%)**
On ten-year cohort		
Sensitivity	76.7 (73.3, 79.8)	77.3 (73.9, 80.4)
Specificity	96.4 (96.1, 96.8)	89.9 (89.3, 90.5)
On 2015-year cohort: with more complete IC data available		
Sensitivity	70.5 (62.9, 77.1)	73.1 (65.6, 79.4)
Specificity	97.1 (96.6, 97.6)	89.7 (88.7, 90.6)

95% confidence intervals are presented in parentheses

^a^The positive pool for sensitivity includes screen-detected positives and two-year ICs, which are relevant for HU

^b^The positive pool for sensitivity includes screen-detected positives and three-year ICs, which are relevant for the UK

Overall, the AI system flagged 2,037 of the 2,310 (88.2%) screen-detected cancers, 111 of the 373 (29.8%) historical ICs (three-year ICs in the UK and two-year ICs in HU), and 177 of 631 (28.1%) cases where cancer was historically detected in the next screening round (3-year screening interval in the UK and 2-year screening interval in HU). In comparison, the historical first reader flagged 2,086 of the 2,310 (90.3%) screen-detected cancers, 26 of the 373 (7.0%) historical ICs, and 41 of the 631 (6.5%) cases where cancer was historically detected in the next screening round.

On the 2015-year cohort, where more complete IC data is available, the AI system flagged 34 of the 92 (37.0%) historical ICs, whereas the historical first reader flagged 4 (4.3%). The AI system also flagged 53 of 198 (26.8%) cases where cancer was historically detected in the next screening round, whereas the historical first reader flagged 9 (4.6%).

### Operational impact

When used as an independent reader in a double reading workflow, the AI system automates the second read. This reduction in the number of human readers was offset by an increased proportion of cases requiring arbitration from 3.3% (3.2%, 3.3%) to 12.3% (12.2%, 12.5%) when using the AI system as an independent reader. These results suggest that applying the AI system would have reduced the number of case assessments requiring human readers by 251,914 over the study period. Assuming that reading time at arbitration may be up to four times greater than the first or second reads, this would amount to decreasing the overall workload between 30.0% and 44.8% across all the mammography equipment vendors and sites when accounting for the indicated increase in arbitration rate (12.3% vs 3.3%). The workload reduction estimates were consistent across mammography equipment vendors and sites, ranging from 38.2% to 46.9% for MK/IMS Giotto, 30.5% to 44.9% for NUH/GE, 24.0% to 43.2% for LTHT/Hologic, and 27.0% to 44.0% for ULH/Siemens.

## Discussion

Many European countries rely on double reading to achieve high cancer detection rates while maintaining low recall rates. The high resource requirements of double reading, however, lead to sustainability pressures under workforce shortages. An AI system that serves as a robust and reliable independent reader in breast cancer screening may help address both clinical and socioeconomic needs, and help make high quality screening more widely available. In this large-scale, multi-vendor, retrospective study we found that a commercially available AI system could be used as an independent reader in the double reading breast cancer screening workflow with robust and consistent performance across different screening environments including different mammography equipment, demographics, geographies, and screening intervals.

Double reading performance with the AI, compared to historical human double reading, demonstrated generalisation across four different mammography equipment vendors and sites (Table 2) and at a geographical level (Table 3), with at least non-inferior recall rate, CDR, sensitivity, specificity, and PPV. Notably, the reduction in the workload between 30% to 44.8% may significantly lighten the pressure on screening services where qualified reading workforce is limited. The effectiveness of AI to serve as an independent reader and to provide workload savings was consistent across different mammography equipment.

It should be noted that cancer detection results (sensitivity, CDR and PPV) in this study are expected to indicate the lower-bound of real-world double reading performance with the AI system, while the comparator human double reading results are exact (for CDR and PPV) or upper-bound (for sensitivity). Due to the retrospective nature of this study, we have missing information on cancers historically not detected, thus skewing the observed historical sensitivity to be higher (or upper bound). At the same time, a subset of these cancers detected by the AI are not recognised in this retrospective setting, unless they were recognised as interval cancers. The consequence is that a number of cancers that would be true positives for the AI are actually recognised as false positives, leading to a skew to lower values in sensitivity, CDR, PPV and specificity for the standalone AI and DR with AI simulation results. The higher observed cancer detection performance on the 2015-year cohort with more complete IC data also supports the expectation that cancer detection performance with the AI is bounded by incomplete IC or missing cancer information.

A second factor that constrains the DR with AI results comes from the use of the historical second reader opinion when the historical arbitration opinion was not available. Historical second readers are expected and observed to have lower performance (particularly in sensitivity, see Table S4 in supplement) than the historical arbitrator since arbitrators have the advantage of being informed with previous reader opinions and often can spend longer time reading arbitration cases. Taking into account the limitations of censorship on positives and the simulation approach, the cancer detection performance of double reading with the AI could potentially be higher in a real-world setting compared to the simulation results in this study. The cancer detection results presented here could present lower-bound estimates which, however, would need to be confirmed in prospective settings.

When assessed on its own, the AI system showed consistent performance across different mammography equipment and found 30% to 37% of historical ICs, indicating that cancer detection could be improved with the aid of the AI system. Only a small portion (11.8% to 24.3%) of the historical ICs detected by the AI system were also detected in the simulated double reading with the AI system. This suggests that the AI may be suitable to additionally serve in alternative workflows, such as a safety net to flag cases not recalled by double reading.

The specificity of the AI system was lower than the historical first human reader, which contributed to increased arbitration in double reading but no increase in actual recalls. The results indicate that using a sensitive AI system may actually help reduce the recall rate rather than increase, subject to the reader behaviour that arises around AI at each site. Overall performance is expected to increase with future improvements of the AI by taking into account the image information available in prior screening rounds.

The contribution of this study is that the AI system’s performance as an independent reader and standalone was evaluated and compared across diverse screening environments at large-scale. Cohorts covered two national screening programmes with a variety of demographic differences and four different mammography equipment vendors. There was some geographic variability in screening performance across the two nations, however, all sites are upheld to their national breast screening programme guidelines for double reading. Our study does not rely on data-construction to approximate a screening cohort or prevalence, but is based on an unenriched cohort where confirmed positives and negatives, and unconfirmed cases were all included. In contrast, small-scale reader studies [22–25, 27] of the past have been employed with enriched cohorts of 320 to 720 cases and no more than two mammography equipment vendors. Larger-scale retrospective evaluations [25, 26, 28, 29] have been employed with 8,805 to 122,969 cases with mostly 1–2 mammography equipment vendors. McKinney et al [25] used data skewed towards a single mammography equipment vendor (> 95%), Salim et al [26] used data from a single mammography equipment vendor, Larsen et al [28] used data from two mammography equipment vendors, and Leibig et al [29] used data from three mammography equipment vendors with a skew towards two (> 91%) and present results across the mammography equipment vendors, but for enriched datasets. Previous reader and retrospective studies [22–27, 29, 30] assessed AI performance utilising cohorts that were enriched for cancer cases and excluded unconfirmed cases, typically the hardest for AI to assess correctly. Instead most apply resampling to approximate a screening prevalence but not necessarily reflect a real-world screening case distribution. This is significant as data-construction can introduce unwanted biases and not including hard unconfirmed cases is likely to result in optimistic performance that does not translate to real-world environments. Across past works, there has been insufficient evidence for the generalisation of AI across heterogeneous clinical environments such as mammography equipment differences and insufficient representative evidence of how AI may perform on real-world populations and in real-world diverse environments. This study presents results for use of AI as an independent reader and standalone across four mammography equipment vendors equally represented, on unenriched data representative of what the AI would process in real-world environments and data from different countries and screening programmes.

The retrospective nature of this evaluation enabled the large-scale assessment of diverse cohorts. However it also implies several inherent limitations. In the double reading simulation with AI, it is assumed that the historical arbitration opinion would remain the same if presented with the AI system’s opinion instead of the historical second reader’s opinion. Prospective studies are required to assess how inclusion of the AI system’s opinion may influence their reading behaviour towards determining a final opinion. As described above, cancer detection with AI is expected to be higher in a real-world setting than what has been measured retrospectively, which needs to be confirmed. Ensuring recall rates are maintained or improved would also be important to confirm prospectively when arbitrators have access to the AI system’s opinions. With more complete IC data in the 2015-year cohort, the point estimates for sensitivity and CDR can be expected to be more representative, but the smaller cohort size resulted in wider confidence intervals. Estimating the impact of incomplete IC data on outcome metrics will be the subject of future work.

The results from the retrospective evaluation suggest that the AI system could be a promising solution when acting as an independent reader in the double reading workflow. In the double reading simulation with AI, the double reading standard of care screening performance was preserved at all relevant screening metrics. The scale and diversity of cohorts and the use of data from multiple mammography equipment vendors indicates that the results may generalise to other screening programmes. Reducing the overall double reading workload can support sustainability and can also enable staff redeployment to alternative activities for the sake of service improvements such as increased patient interaction, more time for training, an extended programme age range, and more focus on complex cases.

## Supplementary Information

**Additional file 1: Table S1.** An assessment of metrics used for evaluating performance with AI in breast screening.** Table S2.** Cases recalled and not recalled by double reading with and without AI – by site/vendor.** Table S3.** Cases recalled and not recalled by standalone AI the historical first reader – by site/vendor.** Table S4.** Cases historically arbitrated or not when the AI and historical first reader agree or disagree.

## Data Availability

The full study protocol and data generated or analysed during the study are available from the corresponding author by request. The imaging datasets are obtained under licences for this study, and are not publicly available.
