# Supplementary material for: Multi-vendor evaluation of artificial intelligence as an independent reader for double reading in breast cancer screening on 275,900 mammograms
Source: BMC Cancer. 2023 May 19;23:460. doi: 10.1186/s12885-023-10890-7 (PMC10197505; doi:10.1186/s12885-023-10890-7)
Supplement: Supplementary file 1 — Additional file 1: Table S1. An assessment of metrics used for evaluating performance with AI in breast screening. Table S2. Cases recalled and not recalled by double reading with and without AI – by site/vendor. Table S3. Cases recalled and not recalled by standalone AI the historical first reader – by site/vendor. Table S4. Cases historically arbitrated or not when the AI and historical first reader agree or disagree. [file 12885_2023_10890_MOESM1_ESM.docx]

**Multi-vendor evaluation of artificial intelligence as an independent reader for double reading in breast cancer screening on 275,900 mammograms**

**Supplementary Information**

# **1) Performance metrics**

Evaluation of an AI system is most reliably assessed on an unenriched representative population. In breast cancer screening, recall rate and cancer detection rate (CDR) are the most informative metrics for evaluating the practical performance of a service. It is worth noting that different screening programmes have different metrics defined and tracked^1-3^. However, for the purposes of assessing AI in breast cancer screening, the characteristics in Table S1 are always relevant.

## Table S1: An assessment of metrics used for evaluating performance with AI in breast screening

| **Metric** | |  | **Strengths** |  | **Limitations** |
| --- | --- | --- | --- | --- | --- |
| **Recall Rate** | | ●  ●  ● | **Common metric tracked in screening programmes**  **Measurable on the entire cohort including unconfirmed cases (not just confirmed positives and confirmed negatives) as not dependent on ground truth definitions**  **The surrogate for specificity that is relevant for screening practice** | **●** | **Only meaningful on unenriched unfiltered cohorts that have the correct screening prevalence** |
| **Cancer**  **Detection**  **Rate (CDR)** | | ●  ●  ● | **Common metric tracked in screening programmes**  **The surrogate for sensitivity that is relevant for screening practice**  **Intuitive, as it increases for the AI with more IC data available** | **●**  **●** | **Dependent on a positivity ground truth definition**  **Dependent on the screening programme (screening interval, performance, prevalence)** |
| Sensitivity | | ●  ● | A standard diagnostic test metric  Independent of prevalence | ●  ●  ● | Dependent on a positivity ground truth definition  Not a standardised screening metric  Decreases with more IC data available |
| Specificity |  | ●  ● | A standard diagnostic test metric  Independent of prevalence | ●    ● | Dependent on a negativity ground truth definition  Not a standardised screening metric |
|  |  |  |  |  |  |
| Positive  Predictive  Value (PPV) |  | ● | A standard diagnostic test metric sometimes used in screening | ●  ● | Depending on the PPV definition used (i.e. over all recalls or over sum of true positives and false positives), can mean an upper or lower bound  Dependent on a positivity ground truth definition and may depend on a negativity ground truth definition |
| Arbitration  Rate |  | ●  ● | Measurable on the entire cohort including unconfirmed cases (not just confirmed positives and confirmed negatives) as not dependent on ground truth definitions  Relevant for operational impact in practice | ● | Implications depend on the nature of the arbitration process which may differ across sites and programmes |

#

# **2) Number of Cases Recalled and Not Recalled**

The number of confirmed negative, confirmed positive, and unconfirmed cases recalled and not recalled by double reading with and without AI and by standalone AI versus the historical first reader are presented in Tables S2 and S3, respectively, by site and mammography equipment vendor.

## Table S2: Cases recalled and not recalled by double reading with and without AI – by site/vendor.

| **A) MK / IMS Giotto**^1^ | | | | |
| --- | --- | --- | --- | --- |
| **Type of case** | **Historical double reading** | | **Double reading (DR) with AI** | |
|  | **Cases Recalled** | **Cases Not Recalled** | **Cases Recalled** | **Cases Not Recalled** |
| On ten-year cohort | | | | |
| Confirmed negative | 710 | 12607 | 564 | 12753 |
| Confirmed positive | 639 | 81 | 630 | 90 |
| Unconfirmed | 6316 | 63057 | 5348 | 64025 |
| On 2015-year cohort: with more complete IC data available | | | | |
| Confirmed negative | 60 | 1372 | 45 | 1387 |
| Confirmed positive | 78 | 11 | 77 | 12 |
| Unconfirmed | 756 | 8185 | 663 | 8278 |
| **B) NUH / GE**^2^ | | | | |
| **Type of case** | **Historical double reading** | | **Double reading (DR) with AI** | |
|  | **Cases Recalled** | **Cases Not Recalled** | **Cases Recalled** | **Cases Not Recalled** |
| On ten-year cohort | | | | |
| Confirmed negative | 454 | 21077 | 445 | 21086 |
| Confirmed positive | 608 | 103 | 594 | 117 |
| Unconfirmed | 865 | 45938 | 913 | 45890 |
| On 2015-year cohort: with more complete IC data available | | | | |
| Confirmed negative | 140 | 6935 | 132 | 6943 |
| Confirmed positive | 88 | 31 | 87 | 32 |
| Unconfirmed | 83 | 3706 | 89 | 3700 |
| **C) LTHT / Hologic**^2^ | | | | |
| **Type of case** | **Historical double reading** | | **Double reading (DR) with AI** | |
|  | **Cases Recalled** | **Cases Not Recalled** | **Cases Recalled** | **Cases Not Recalled** |
| On ten-year cohort | | | | |
| Confirmed negative | 896 | 21049 | 877 | 21068 |
| Confirmed positive | 533 | 79 | 519 | 93 |
| Unconfirmed | 1864 | 40224 | 1869 | 40219 |
| On 2015-year cohort: with more complete IC data available | | | | |
| Confirmed negative | 200 | 5455 | 191 | 5464 |
| Confirmed positive | 82 | 11 | 81 | 12 |
| Unconfirmed | 181 | 4788 | 170 | 4799 |
| **D) ULH / Siemens**^2^ | | | | |
| **Type of case** | **Historical double reading** | | **Double reading (DR) with AI** | |
|  | **Cases Recalled** | **Cases Not Recalled** | **Cases Recalled** | **Cases Not Recalled** |
| On ten-year cohort | | | | |
| Confirmed negative | 256 | 9449 | 246 | 9459 |
| Confirmed positive | 548 | 92 | 534 | 106 |
| Unconfirmed | 1329 | 47126 | 1309 | 47146 |
| On 2015-year cohort: with more complete IC data available | | | | |
| Confirmed negative | 102 | 4090 | 97 | 4095 |
| Confirmed positive | 121 | 35 | 119 | 37 |
| Unconfirmed | 239 | 8926 | 239 | 8926 |

1. Confirmed positives include screen-detected positives and two-year ICs, which are relevant for HU.
2. Confirmed positives include screen-detected positives and three-year ICs, which are relevant for the UK.

## Table S3: Cases recalled and not recalled by standalone AI the historical first reader – by site/vendor.

| **A) MK / IMS Giotto**^1^ | | | | |
| --- | --- | --- | --- | --- |
| **Type of case** | **Historical first reader** | | **Standalone AI** | |
|  | **Cases Recalled** | **Cases Not Recalled** | **Cases Recalled** | **Cases Not Recalled** |
| On ten-year cohort | | | | |
| Confirmed negative | 619 | 12698 | 523 | 12794 |
| Confirmed positive | 572 | 148 | 617 | 103 |
| Unconfirmed | 5280 | 64093 | 5134 | 64239 |
| On 2015-year cohort: with more complete IC data available | | | | |
| Confirmed negative | 50 | 1382 | 55 | 1377 |
| Confirmed positive | 73 | 16 | 76 | 13 |
| Unconfirmed | 656 | 8285 | 570 | 8371 |
| **B) NUH / GE**^2^ | | | | |
| **Type of case** | **Historical first reader** | | **Standalone AI** | |
|  | **Cases Recalled** | **Cases Not Recalled** | **Cases Recalled** | **Cases Not Recalled** |
| On ten-year cohort | | | | |
| Confirmed negative | 585 | 20946 | 2233 | 19298 |
| Confirmed positive | 553 | 158 | 547 | 164 |
| Unconfirmed | 1373 | 45430 | 4925 | 41878 |
| On 2015-year cohort: with more complete IC data available | | | | |
| Confirmed negative | 201 | 6874 | 666 | 6409 |
| Confirmed positive | 80 | 39 | 86 | 33 |
| Unconfirmed | 114 | 3675 | 434 | 3355 |
| **C) LTHT / Hologic**^2^ | | | | |
| **Type of case** | **Historical first reader** | | **Standalone AI** | |
|  | **Cases Recalled** | **Cases Not Recalled** | **Cases Recalled** | **Cases Not Recalled** |
| On ten-year cohort | | | | |
| Confirmed negative | 1101 | 20844 | 2362 | 19583 |
| Confirmed positive | 496 | 116 | 489 | 123 |
| Unconfirmed | 2389 | 39699 | 6324 | 35764 |
| On 2015-year cohort: with more complete IC data available | | | | |
| Confirmed negative | 243 | 5412 | 603 | 5052 |
| Confirmed positive | 77 | 16 | 79 | 14 |
| Unconfirmed | 211 | 4758 | 597 | 4372 |
| **D) ULH / Siemens**^2^ | | | | |
| **Type of case** | **Historical first reader** | | **Standalone AI** | |
|  | **Cases Recalled** | **Cases Not Recalled** | **Cases Recalled** | **Cases Not Recalled** |
| On ten-year cohort | | | | |
| Confirmed negative | 345 | 9360 | 982 | 8723 |
| Confirmed positive | 491 | 149 | 495 | 145 |
| Unconfirmed | 1656 | 46799 | 6262 | 42193 |
| On 2015-year cohort: with more complete IC data available | | | | |
| Confirmed negative | 120 | 4072 | 433 | 3759 |
| Confirmed positive | 110 | 46 | 114 | 42 |
| Unconfirmed | 284 | 8881 | 1043 | 8122 |

1. Confirmed positives include screen-detected positives and two-year ICs, which are relevant for HU.
2. Confirmed positives include screen-detected positives and three-year ICs, which are relevant for the UK.

#

#

# **3) Portion of cases historically arbitrated or not**

The occurrence of cases historically arbitrated or historically not arbitrated when the AI and the historical first reader agree or disagree are presented in Table S4. Additionally, the performance of the historical arbitrator and historical second reader are presented for the set of cases when the AI and historical first reader agree and disagree in Table S4.

When the AI and first reader do not agree, on average the historical second reader is more sensitive and less specific on historically arbitrated cases compared to historically not arbitrated cases. The historical arbitrator is even more sensitive and even less specific than the historical second reader on historically arbitrated cases.

When the AI and first reader do agree, on average the historical second reader is significantly less sensitive and less specific on historically arbitrated cases compared to historically not arbitrated cases. The historical arbitrator, however, demonstrates similar performance trends as when the AI and first reader do not agree.

## Table S4: Cases historically arbitrated or not when the AI and historical first reader agree or disagree.

|  | | **AI and First Reader Agree** | | | | | **AI and the First Reader Do Not Agree** | | | | |
| --- | --- | --- | --- | --- | --- | --- | --- | --- | --- | --- | --- |
|  |  | **Cases** | **Historical Arbitrator** | | **Second Reader** | | **Cases** | **Historical Arbitrator** | | **Second Reader** | |
|  |  | Value (%) | Sens (%) | Spec (%) | Sens (%) | Spec (%) | Value (%) | Sens (%) | Spec (%) | Sens (%) | Spec (%) |
| On ten-year cohort across all sites | | | | | | | | | | | |
| **Historically Arbitrated** | | 4436 (1.8%) | 94.3% | 56.3% | 48.9% | 24.4% | 4943 (14.9%) | 96.2% | 66.3% | 85.7% | 78.9% |
| **Historically Not Arbitrated** | | 238273 (98.2%) |  |  | 87.8% | 99.0% | 28248 (85.1%) |  |  | 66.7% | 80.2% |
| On ten-year cohort per region | | | | | | | | | | | |
| **Historically Arbitrated** | **UK** | 2588 | 93.1% | 66.3% | 58.3% | 27.6% | 3186 | 95.4% | 76.3% | 86.2% | 78.1% |
|  | **HU** | 1848 | 97.9% | 26.5% | 20.8% | 14.8% | 1757 | 98.6% | 36.5% | 84.1% | 81.6% |
| **Historically Not Arbitrated** | **UK** | 163816 |  |  | 87.5% | 99.0% | 22900 |  |  | 70.3% | 87.9% |
|  | **HU** | 74457 |  |  | 88.8% | 99.0% | 5348 |  |  | 55.9% | 57.0% |

Sens = Sensitivity; Spec = specificity

# **4) Supplementary Information References**

1. National Health Institutes England, Public Health England. NHS Breast screening programme screening standards valid for data collected from 1 April 2017. 2021. https://www.gov.uk/government/publications/breast-screening-consolidated-programmestandards/nhs-breast-screening-programme-screening-standards-valid-for-data-collected-from-1april-2017.
2. Radiológiai Szakmai Kollégium (Professional Association of Radiologists, Hungary). Az Egészségügyi Minisztérium szakmai protokollja mammográfiás emlőszűrésről és a korai emlőrák diagnosztikájáról. Egészségügyi Közlöny. 2008.05.28; LVIII/10 (2990-3012)
3. Perry N, Broeders M, de Wolf C, et al. European guidelines for quality assurance in breast cancer screening and diagnosis. Fourth edition—summary document. Annals of Oncology. 2008;19(4):614-22.
